# All-Food-Seq (AFS): a quantifiable screen for species in biological samples by deep DNA sequencing

**DOI:** 10.1186/1471-2164-15-639

**Published:** 2014-07-31

**Authors:** Fabian Ripp, Christopher Felix Krombholz, Yongchao Liu, Mathias Weber, Anne Schäfer, Bertil Schmidt, Rene Köppel, Thomas Hankeln

**Affiliations:** Institute of Molecular Genetics, Johannes Gutenberg University Mainz, D55099 Mainz, Germany; Institute of Computer Science, Johannes Gutenberg University Mainz, D55099 Mainz, Germany; Official Food Control Authority of the Canton Zürich, Zürich, Switzerland

**Keywords:** Illumina, Next-generation sequencing, Real-time PCR, Species identification, Metagenomics

## Abstract

**Background:**

DNA-based methods like PCR efficiently identify and quantify the taxon composition of complex biological materials, but are limited to detecting species targeted by the choice of the primer assay. We show here how untargeted deep sequencing of foodstuff total genomic DNA, followed by bioinformatic analysis of sequence reads, facilitates highly accurate identification of species from all kingdoms of life, at the same time enabling quantitative measurement of the main ingredients and detection of unanticipated food components.

**Results:**

Sequence data simulation and real-case Illumina sequencing of DNA from reference sausages composed of mammalian (pig, cow, horse, sheep) and avian (chicken, turkey) species are able to quantify material correctly at the 1% discrimination level via a read counting approach. An additional metagenomic step facilitates identification of traces from animal, plant and microbial DNA including unexpected species, which is prospectively important for the detection of allergens and pathogens.

**Conclusions:**

Our data suggest that deep sequencing of total genomic DNA from samples of heterogeneous taxon composition promises to be a valuable screening tool for reference species identification and quantification in biosurveillance applications like food testing, potentially alleviating some of the problems in taxon representation and quantification associated with targeted PCR-based approaches.

**Electronic supplementary material:**

The online version of this article (doi:10.1186/1471-2164-15-639) contains supplementary material, which is available to authorized users.

## Background

Biosurveillance is a necessary task to monitor food for human consumption and pharmaceutical drugs, subsumed as “biologicals”, which typically consist of complex mixtures of processed biological material. Since the species origin of such products is often unclear, there is a concern about fraud, health risks and violation of ethical/religious principles, as best illustrated by the 2013 European horse meat case (for a further bizarre example see: http://www.bbc.com/news/world-asia-17980177). Therefore, food and drug legislation demands producers to provide a proper declaration of ingredients, e.g. by naming species components of food products [1, 2]. To ensure correct declaration, accurate and efficient analytical methods of foodstuff analysis have been developed, mostly based on the detection of species on the DNA level by PCR [3, 4]. Such DNA-based tests are considered superior to protein-based methods, when processed material has to be analyzed [5]. Real-time PCR assays, often based on fast-evolving gene regions from the abundant mitochondrial genome, now facilitate a multiplex detection of many bird, fish and mammalian taxa [6–9]. Such assays sometimes even allow for discrimination of taxa as closely related as cow and water buffalo [10]. The main shortcoming of PCR-based detection methods is, however, that they inherently target only DNA from species to which the PCR primers bind efficiently. This caveat also holds true in principle for barcoding methods that rely on the PCR amplification and subsequent massively parallel next-generation sequencing of amplicons from variable genomic or cell organelle DNA regions (e.g. 16S rDNA, rDNA-ITS or mitochondrial COI [11]). Barcoding methods have been shown to very efficiently identify taxa within environmental or food-derived metagenomic samples in a qualitative way [12–15], but require separate assays to address the different domains of life. In addition, quantification of components by barcode sequencing has proven problematic due to taxonomic biases induced by the varying primer binding efficiencies across taxa ([13–16]; and references therein). Species quantification by sequencing of organellar PCR amplicons is also critical, as the absolute number of mitochondrial genomes per cell is highly fluctuating already between different tissues (e.g. eight-fold within different human cell types [17]). In contrast, sequence analysis of total genomic DNA isolated from food offers in principle the possibility to detect species in a totally unbiased way, enabling e.g. the detection of fraud through admixture of undeclared ‘exotic’ taxa or the presence of health risks by microbial contamination [4]. In the field of gene expression analysis, NGS sequencing facilitates a robust quantitative analysis of RNA molecules through digitally counting sequence reads obtained from the cDNA population of a tissue [18, 19]. The sensitivity and dynamic range of read counting equals or supersedes other quantitative DNA analytical methods like microarrays or SAGE [20, 21]. From a technical perspective, species identification based on the whole-genome sequencing should also be feasible since the large, non-protein-coding part of eucaryotic genomes evolves rather quickly and strongly conserved gene exons constitute only a minor proportion, e.g. roughly 1.5% of a mammalian genome [22]. Therefore, even closely related food-relevant taxa such as goat and sheep or turkey and chicken should be distinguishable in a total genomic comparison. Intraspecific polymorphism in foodstuff species ranges between 0.5 to 5 nucleotides per 1,000 bp in horse, swine and chicken, respectively [23–26], which should not substantially affect species discrimination.

Here we show that deep sequencing of total DNA derived from foodstuff material can readily identify and quantify species components with high accuracy by a single experimental assay. Sequence reads are assigned to species by mapping [27, 28] to publicly available reference genome sequences, which steadily grow in number, as exemplified by the Genome10k Project (https://genome10k.soe.ucsc.edu). At the same time, reads of “unexpected” species origin are readily detected by a metagenomic analysis based on DNA sequence database searching.

## Methods

### The bioinformatics pipeline

**Figure 1:**
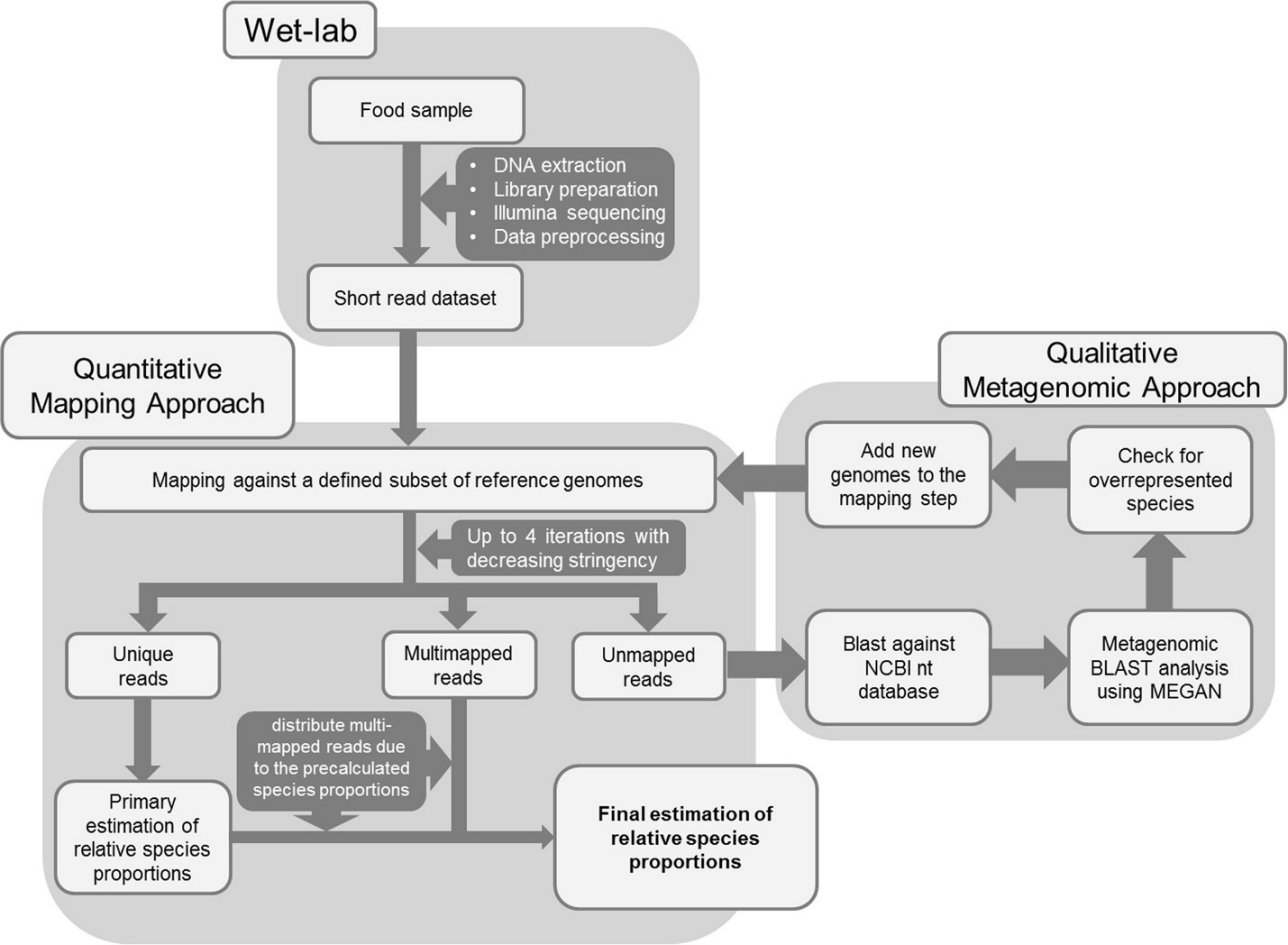
**Outline of the AFS pipeline.**

Sequence reads of 100 bp, either obtained by simulation (see below) or by Illumina sequencing of DNA from sausage meat (see below), were initially mapped against reference genomes using the algorithms BWA (V 0.7.0; [29]) or CUSHAW [30] resulting in a SAM file for each mapping. Reference genomes in our pilot analysis comprised the species *Bos taurus*, *Bubalus bubalis, Equus caballus, Escherichia coli, Gallus gallus, Glycine max, Homo sapiens, Listeria seeligeri, Meleagris gallopavo, Mus musculus, Neisseria gonorrhoeae, Oryctolagus cuniculus, Oryza sativa, Ovis aries, Rattus norvegicus, Shigella boydii, Sus scrofa, Triticum aestivum* and *Zea mays* (for details see Additional file 1). Reference genome taxa were mostly chosen either because of their foodstuff relevance or matches obtained in the metagenomic analyses step of our pipeline. Others (like human or rat) were primarily included to serve as negative controls to judge the extent of false positive read assignments. It is clear that for a broader screening many more reference genomes could have been used. The practical upper limit for the number of reference genomes clearly depends on computer power and scales linearly with time. The BWA mappings were executed by allowing 0, 1, 2 or 3 mismatches, depending on the respective approach (see below). For the downstream analysis of the mapping results we utilized SAMtools (V 0.1.18; [31]) and a set of self-implemented Perl scripts.After the mapping step, we identified three sets of sequence reads (Figure 1). The first set contained reads mapping to just one genome (“unique reads”). Assigning these reads to a genome and quantifying them by counting was a straightforward task. More challenging were reads, which covered conserved sequence regions within genomes and therefore simultaneously hit at least two different genomes (“multi-mapped reads”), even under conditions of the highest mapping stringency. Since these conserved reads cannot be assigned with any certainty to one specific genome, we distributed them to the respective candidate genomes in the proportion previously calculated from the unique reads. By this means, the multi-mapped reads could additively be used to improve the values of the quantitative analysis.

A 3^rd^ category, so-called “unmapped reads”, were collected and forwarded to up to three further rounds of mapping, each of which allows one more mismatch than the previous round (i.e. in round 4 we had a matching stringency of 97%). We then calculated the proportions of species material from all reads, which were unambiguously assigned at this step. To account for the different quality (i.e. completeness) of the reference genomes, as indicated by different numbers of positions denoted by Ns in the genome drafts, our initial quantitative estimates were corrected by a genome quality factor *f* = (*n* + *c*)/*c*, where *n* is the number of ambiguous nucleotides and *c* is the total number of nucleotides in the reference genome. Further normalization should in principle be necessary to adjust for largely different genome sizes, e.g. when comparing birds and mammals which differ roughly 3-fold in DNA content [32, 33]. However, our quantification of a sample containing avian material (Additional file 2) indicated that such normalization might be unnecessary, possibly due to the correlation of smaller genome size with a smaller nucleus and cell size [34] leading to a compensatory denser packaging of cells per gram avian tissue.

In our pipeline, we subsequently tried to identify the origin of still unmapped reads by BLASTN (V 2.2.25) database searching [35] against the NCBI nucleotide database (nr/nt). Since our query sequences were short 100 bp reads, we used a word size of 11, set the BLAST e-value to 100 according to MEGAN’s “how to use BLAST” tutorial [36], and accepted the best three hits for further analysis. Furthermore, we set BLAST’s “*–I*” option to add the *gi* number to the default BLAST output files. Otherwise, default BLASTN settings were used. BLASTN results were then visualized by the metagenomic analysis tool MEGAN4 (V 4.70.4). This tool parses BLAST output files and assigns the results to species or, if this is not possible, taxonomic groups of higher rank according to the NCBI taxonomy database. To filter out false-positives, caused by low complexity repeats (e.g. microsatellites) or highly conserved regions, we set MEGAN’s LCA parameters to *Min complexity* = 0.44, *Min Score* = 75.0, *Top percent* = 1.0 and turned on the percent identity filter. To limit the analysis to the most relevant results, taxa were somewhat arbitrarily only accepted for visualization in our pilot study, if more than 50 reads were assigned to this taxon. BLAST results were then visualized as a phylogenetic tree and quantified using Excel. For species attracting more than a threshold number of the unmapped reads in the BLAST step, a return to the read mapping procedure would be reasonable to infer more exactly the proportion of this taxon. This, of course, requires the availability of the respective reference genome, the list of which is gradually increasing.

### Dataset simulation and calculation

As a proof of principle, we simulated records of Illumina sequence data by randomly extracting 100 bp long sequences from downloaded genome sequences, which were subsequently tagged by their origin (Additional file 1). Random errors were introduced into the simulated reads at a 1% rate. Next, we compiled mixed datasets for testing the read-mapping pipeline, by randomly sampling subsets of these simulated sequence reads. For simplicity at this testing stage, we did not perform iterative mapping at different stringencies, but allowed only one mismatch in the mapping process. We also did not apply the genome quality factor.

### Illumina sequencing of DNA from a sausage calibration sample

Total genomic DNA was extracted from 200 mg of the homogenized calibration sausages “KalD” (type “boiled sausage”) [37] and “KLyoA” (type “Lyoner”) [9] by the Wizard Plus Miniprep DNA purification system (Promega, Madison, USA). DNA was eluted in 50 μl elution buffer according to the supplier's manual. Illumina sequencing library preparation was conducted on 1.5 μg of total DNA by StarSEQ (Mainz, Germany) using the TruSeq DNA Sample Preparation Kit v.2 (Illumina, San Diego, USA). Sequencing was performed on an Illumina HiSeq 2000 instrument (100 bp paired-end reads) for KalD and on a MiSeq instrument (50 bp single reads) for KLyoA. We used the FASTX toolkit (http://hannonlab.cshl.edu/fastx_toolkit/index.html) for adapter clipping and quality filtering. Reads shorter than 50 bp (KalD) or 20 bp (KLyoA) were discarded.

### Hardware for bioinformatical analyses

For the sake of speed, mappings using BWA were preferentially performed on one node (containing 4 CPUs with 16 cores each running at 2.1 GHz) of the Mogon Linux-Cluster at University of Mainz. Each iterative mapping (4 rounds, 0 to 3 mismatches) with 1 mio paired-end reads took about 45 minutes. Mapping on a standard PC (4 × 2.67 GHz, 16 GB RAM) consumed 3 hrs of time using 12 reference genomes (30 Gbp size) and 100,000 reads. The BLAST steps of the pipeline were run on the University of Mainz Central Computing Linux-Cluster Lc2 (Suse Linux Enterprise Server 10 SP2, 132 nodes containing 2 CPUs with 8 cores each running at 2.7 GHz). Blast requests (single-threaded) were split up to 1000 separate jobs, which reduced runtime to less than 2 hours for 200,000 queries. The MEGAN program was subsequently run on a standard personal computer (PC) with 8 GB RAM and Windows OS.

## Results and discussion

### Read mapping facilitates exact quantification of DNA from diverse species

To test if high-throughput genomic DNA sequencing was able to accurately determine the proportions of foodstuff components, we initially simulated two sets of sequencing records (Table 1 and 2). The simulated sequence data was based on randomly sampled sub-sequences of public available genomes with randomly introduced errors.

**Table 1:** **Mapping results from simulated datasets**

Species	Reads assigned	Proportion [%]	Target value [%]	Difference abs. [%]	Difference rel. [%]
**Cattle**	26,555	2.95	3	0.05	1.67
**Horse**	224,077	24.91	25	0.09	0.36
**Human**	8,969	1.00	1	0.00	0.00
**Pig**	89,421	9.94	10	0.06	0.60
**Rat**	9,042	1.01	1	0.01	1.00
**Sheep**	541,432	60.19	60	0.19	0.32

Simulated quantification of sequence reads obtained from six different genomes using the AFS pipeline. “Difference abs.” shows the difference between the proportions of reads, as determined by AFS (“proportion”), relative to the expected amounts existing in the sample (“target value”). “Difference rel.” is calculated by dividing “Difference abs.” by the expected proportion value.

**Table 2:** **Effect of reference genome choice**

Species	Target value	Proportion without ***E. coli***genome [%]	Proportion with ***E. coli***genome [%]	Difference before [%]	Difference after [%]
**Cattle**	58.82	61.81	58.53	2.99	0.29
**E.coli**	5.89	0.00	5.86	5.89	0.02
**Pig**	11.76	13.00	11.74	1.24	0.02
**Sheep**	23.53	25.19	23.86	1.66	0.33

Simulation demonstrates the effect of choosing the adequate genomes for quantification by AFS. Initially, the *E. coli* reference genome was omitted in the mapping step. After observing *E. coli* reads in the metagenomic analysis, its genome was added to the mapping procedure, and the species proportions were now recovered with much higher accuracy.

Dataset 1 consists of one million reads derived from six mammalian species (Table 1). After running our pipeline on this dataset, the proportions of reads assigned to the respective reference genomes mirrored the sample compounds with high accuracy. The artificial dataset contained sequences present in rather high quantities (60% for sheep) and low amounts (1% for human and rat) indicating that the method worked over a broad range of proportions. We achieved absolute differences between assigned reads and input read numbers of 0 to 0.19% (Table 1). The maximum relative difference (% absolute difference/% input DNA) was 1.67%. We also checked the accuracy of the mapping process by tracing the identity of the uniquely mapped reads (represented by different file paths). The mapping accuracy turned out to be better than 99.9% (cattle).

Simulation dataset 2 comprises 850 K reads, mixed at uneven proportions from three mammalian species (cattle, pig, sheep) and the bacterium *E. coli* (Table 2). This dataset was created to check if the method was able to detect signals of “unexpected” species, which would possibly not have a reference genome included in the initial mapping step. The sample was therefore initially run through the pipeline without mapping against bacterial genomes. As a result, all *E. coli* reads were passed on to the metagenomic BLAST/MEGAN step. By this database searching routine, 34,944 of 131,683 unmapped reads (=26.54%) were identified as possible *E. coli* signals. According to our pipeline rationale, the strong *E. coli* signal prompted us to add the *E. coli* genome to the mapping process and to run the pipeline again to achieve a better quantitative estimate of the bacterial reads. In fact, the proportion of *E. coli* reads was now determined at high accuracy with only 0.02% deviation from the real input value (Table 2). Meanwhile, the correct assignment of the *E. coli* reads improved the overall quantitative estimates for all the other species components.

These (and others, not reported) results of our simulation study proved the general feasibility of quantitative species identification through deep sequence analysis of total genomic DNA. Quantification was most exact for the read mapping process implemented in AFS, which however requires the availability of a reference genome. Given that vertebrate (and many other) species will soon be sequenced by the thousands, this requirement will not set a limiting condition on the method itself.

For identifying unexpected species, the application of a less stringent metagenomic search tool, based on a BLAST database search followed by visualization via MEGAN, also proved successful. However, our results for dataset 2 suggested that the mere evaluation of BLAST/MEGAN results would not facilitate an accurate quantitative measurement of read numbers, which is only possible via the more stringently working read mapping algorithms. It should also be stressed that the results of the metagenomic working step entirely depend on the completeness of the sequence database chosen for searching and on the representation of a particular species within a database partition. In addition, one should be cautious of erroneous annotations within public databases [38].

However promising the results of the simulation data analysis appeared, they clearly represented an idealized situation, since we obviously obtained the simulated reads from the very same genomes to which they were mapped thereafter. Hence, we conducted an analysis using real data.

### Illumina sequencing of a DNA sample from calibration sausage material

Illumina sequencing was performed on DNA obtained from sausage material, which previously had been designed and produced as calibration sample for qPCR-based approaches to species identification (KalD, [37], KLyoA [9]). The sample KalD, on which we focused our most detailed analysis, contained material from four mammalian species (35% cattle, 1% horse, 9% pig, 55% sheep). These mammalian taxa feature a minimal interspecific nucleotide divergence at the level of synonymous sites within genes of 7% (for sheep-cattle; [39]), which is most probably exceeded by neutral non-genic sites. In addition, the sausage sample contained admixtures of 11 different plant allergens at varying amounts (R. Köppel, unpublished data; Additional file 3).

After quality filtering, we obtained 2 × 16 million 100 bp paired-end reads. Encouraged by the previous simulations, subsets of only 2 × 500 K (=1 mio) randomly selected paired-end reads were used for further analysis. To account for a possible trade-off between the specificity of taxon identification and a maximally exact quantification of reads, we devised two different mapping strategies. When maximum specificity was the prime goal (“AFS-spec”), we did not allow any mismatches during read mapping, and thus performed only a single mapping step with the highest stringency. In addition, we disabled the Smith-Waterman alignment option in BWA because it lowers the mapping stringency for a paired-end read when rescuing a read from its aligned mate. The second strategy (“AFS-quant”) aimed at best quantitative results. To this end, we performed an iterative read-mapping starting with a stringency of 0 and ending with 3 mismatches.

For both strategies, the n = 3 repetitions produced highly similar results, as evidenced by low standard deviations (Table 3). The AFS-quant approach delivered a highly accurate quantification of meat components in sausage KalD, as exemplified by the value of 54.8% DNA versus 55% (w/w) meat for sheep (Table 3). Absolute differences between the DNA proportions and the meat proportions ranged from 0.24 to 1.79%, showing that species quantification can be achieved at the 1% discrimination level. The highest divergence (1.79%) was observed for pig and can be explained by the use of lard tissue [40], which presumably contains less cells and thus DNA than e.g. muscle tissue. In this respect, AFS behaves in the same well-known matrix-dependent way as other DNA-based detection methods [40], and the definition of normalization values for typical ingredients and production recipes should alleviate this problem also for AFS.

**Table 3:** **Mapping results for the reference sausage KalD**

Species	Target value [%]	Proportion [%]		Difference abs. [%]		Difference rel. [%]	
		AFS-quant	AFS-spec	AFS-quant	AFS-spec	AFS-quant	AFS-spec
**Cattle**	35	36.05 ± 0.04	41.16 ± 0.02	1.05 ± 0.04	6.16 ± 0.02	3 ± 0.11	17.6 ± 0.03
**Horse**	1	1.27 ± 0.01	1.45 ± 0.01	0.28 ± 0.01	0.45 ± 0.01	27.67 ± 0.67	45 ± 1
**Pig**	9	7.22 ± 0.05	7.59 ± 0.09	1.79 ± 0.05	1.41 ± 0.09	19.85 ± 0.48	15.67 ± 1
**Sheep**	55	54.76 ± 0.09	49.71 ± 0.08	0.24 ± 0.09	5.29 ± 0.08	0.44 ± 0.17	9.62 ± 0.15
**Waterbuffalo**	0	0.64 ± 0.03	0.07 ± 0	0.64 ± 0.03	0.07 ± 0	n.a.	n.a.
Total	100			4 ± 0.1	13.38 ± 0.04		

Quantitative species analysis obtained by Illumina sequencing of DNA from the “KalD” reference sausage [37]. The AFS-quant and AFS-spec approaches (see text for details) were compared. Each dataset tested contained 1 mio of paired-end sequence reads, randomly selected from a larger dataset. Three different sub-datasets (1 mio reads each) were analyzed and mean values plus standard deviations are displayed. “Difference abs.” shows the difference between the proportion of reads as determined by AFS (“proportion”) relative to the expected amounts existing in the sample (“target value”). “Difference rel.” is calculated by dividing “Difference abs.” by the expected proportion value.

To infer the specificity of the mapping procedure we included the reference genome sequence of water buffalo, which belongs to the same subfamily (*Bovinae*) as cattle. AFS-quant detected a false-positive proportion of 0.64% DNA reads in the buffalo genome (Table 3), which probably represent sequences strongly conserved between the two bovines. The more stringent AFS-spec approach was able to reduce the false-positive rate of “buffalo reads” substantially to 0.07%, but only at the expense of a markedly diminished accuracy for quantification of the real meat components (Table 3). To demonstrate the broader applicability of the AFS-quant approach we sequenced and quantified the main ingredients of the KLyoA sausage sample, which contains 0.5% chicken and 5.5% turkey on a background of pig and cattle meat (Additional file 2). The avian components were determined as accurate as the mammalian ones.

We conclude that the AFS-quant strategy delivers the most accurate quantitative species determination. We note that the AFS quantification results are equal to or sometimes even better than species analyses performed by quantitative PCR on the same sausage material [37]. AFS still contains a very low risk of obtaining false-positive matches to closely related species. Clearly, further case studies with other species pairs like horse-donkey, which diverged only 2.4 million years ago [41], have to be conducted to generalize our conclusions. As a screening procedure, AFS performance is only limited by the number of reference genomes available. Offering both, a qualitative and quantitative result, deep sequencing of total genomic DNA appears as an excellent alternative to microarray-based screening methods for species identification [42] or sequencing of PCR-based barcode amplicons [12, 13, 15].

### Detection of “unexpected” species via metagenomic analysis

**Figure 2:**
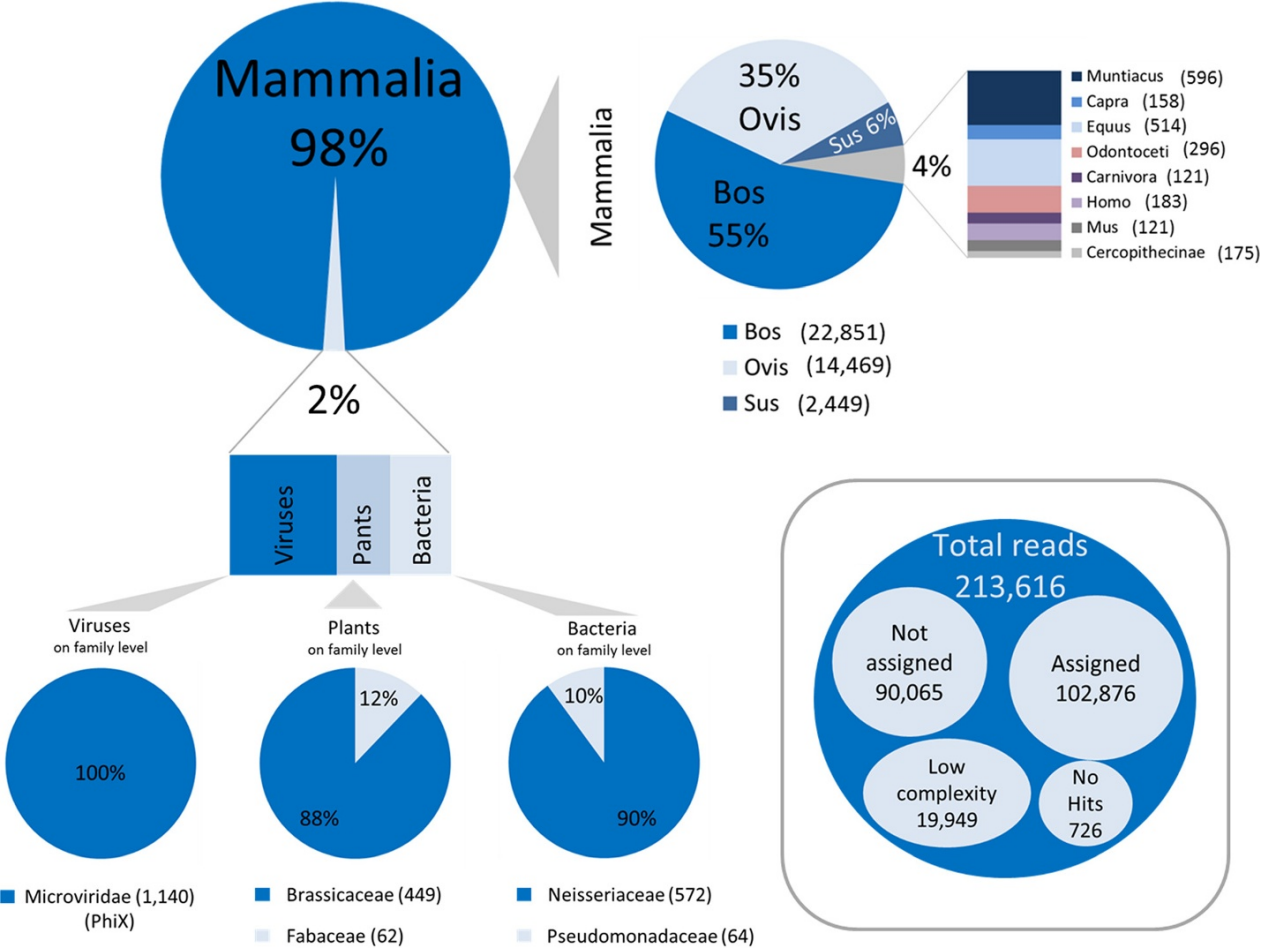
**Metagenomic analysis of unmapped reads.** Results of the metagenomic analysis of sequence reads obtained from the KalD reference sausage. The global result of the BLAST/MEGAN step is shown in the box (grey frame). A more detailed classification of matches is displayed for mammals, viruses, bacteria and plants.

DNA reads which do not map to the selection of reference genomes will be passed over to the BLAST/MEGAN annotation procedure in AFS. The one-million-read datasets obtained from the KalD sausage each produced more than 200 K of unmapped reads (Figure 2). Roughly half of these reads could successfully be assigned to a species or higher ranked taxon. The other half was represented by two classes: (i) low-complexity repetitive DNA (e.g. microsatellites) which is present in almost all genomes and thus cannot be assigned unequivocally; and (ii) un-assignable reads which either did not match an entry in the chosen database or did not meet the stringent MEGAN criteria applied. Clearly, the choice of different specialized databases and perhaps less stringent match criteria has the potential of reducing this portion.

The ca. 100 K reads that were taxonomically assigned by BLAST/MEGAN originated in their vast majority (98%) from mammals (Figure 2). Of those mammalian hits, 96% were annotated as cattle, sheep, pig and horse (i.e. those taxa which formed the sausage). Close inspection of these sequences revealed that they predominantly represented centromeric satellite DNA. This sequence class is usually not represented in genome reference sequences, explaining that the corresponding reads could not be assigned in the mapping step. The observed species proportions of the satellite DNA reads somewhat surprisingly did not match the meat proportions for cattle and sheep. A reason could be that centromeric DNA, which is an inherently unstable component of eucaryotic genomes [43], is present in different amounts in the heterochromatin of sheep and cattle chromosomes, making its use for quantification purposes problematic.

Among the reads of mammalian origins, we further recorded hits to several bovine-related taxa like the muntjac, goat or whales (Figure 2), which separated from bovines 25, 30 and 56 million years ago, respectively (http://www.timetree.org). We could show by an analysis using the tool REPEATMASKER (http://www.repeatmasker.org/) that these reads most often belonged to transposable elements (MIRs, LINEs, ERVs, DNA transposons) which show sequence conservation across this clade. Surprisingly, we also found ~500 matches to human, cercopithecan primates and mouse. Inspection of these BLAST hits revealed that they also contained interspersed repeats. However, in humans and monkeys, none of those reads corresponded to the primate-specific Alu element family. We are thus rather sure that neither goat or whale nor traces of human, monkey or mouse DNA are present in the sample. At the same time, this issue demonstrates that expert interpretation of BLAST results is required, which is by no means a simple task.

Beyond Mammalia, BLAST/MEGAN suggested the presence of viral, bacterial and plant sequences in the sausage DNA (Figure 2). Viral DNA, all belonging to bacteriophage PhiX174, was easily explained since this DNA is usually applied for technical reasons as a calibrator in Illumina sequencing (http://res.illumina.com/documents/products/technotes/technote_phixcontrolv3.pdf). Several hundred bacterial reads were detected, mostly originating from the human-pathogenic species *Neisseria gonorrhoeae* (n = 572 reads) or from *Pseudomonadales* (n = 64 reads), with *Pseudomonas fluorescens* as an often annotated species (n = 45 reads). The latter is notably present e.g. in deteriorating milk and meat products [44]. While the small numbers of *P. fluorescens* reads can be taken as an indicator of beginning food spoilage, the finding of *Neisseria* reads tells a very important cautionary tale in metagenomic analysis. After adding the respective genome [45] to the mapping process, presumed *N. gonorrhoaea* DNA was detected at an amount of 0.04%. Knowing that there should not be *any N. gonorrhoeae* material in the sample, we investigated this result further. By mapping all 32 million reads of our initial dataset to the *N. gonorrhoeae* genome, we obtained matches exclusively located in ten genomic regions, each shorter than 700 bp, where read coverage was extremely high (up to 5200-fold). These regions were extracted from the *N. gonorrhoeae* genome and analyzed by BLAST against the NCBI nucleotide database, thereby revealing strong homology of these parts to ruminant sequence entries (data not shown). In addition, mapping the sausage reads to other available *N. gonorrhoeae* genomes sequences did not produce any matches. We thus question the quality of the *N. gonorrhoeae* strain TCDC-NG08107 genome and recommend using it with high caution. In general, this points out that the annotation quality of database entries is of prime importance to species diagnosis.

Since meat products often contain plant material, the metagenomic analysis on the plant spectrum is of special interest. In fact, the sausage contained admixtures of 11 plant species (Additional file 3) to enable its use in the development of allergen detection methods. The most prominent spiked-in ingredients were lupine (*Lupinus spec.),* walnut (*Juglans regia*), hazelnut (*Corylus avellana*) and mustard (*Brassica spec.).* We detected 661 plant hits, which were assigned to a total of 33 plant families. Amongst those families, *Brassicaceae* (mustard) dominated with 449 hits, followed by *Fabaceae* (lupine, peanut, soy) with 62 hits (Figure 2; Additional file 3). All other plant ingredients received only from 1 to 17 BLAST hits. These numbers of database matches did not correlate with the amount of spiked-in plant material, illustrating that the current BLAST/MEGAN routine is by no means quantitative. A probable reason is the unbalanced representation of sequence entries for the different taxa in the database (data not shown). This can be overcome in future by the production of reference genomes for all major food- and allergenicity-relevant species. In addition, as expected for a DNA-based method, the quantification result will heavily depend on the efficiency of DNA recovery from the food matrix. Of all plant allergens tested, only the genome of soy (*Glycine max*) is publicly available and was thus included in the AFS read mapping step. We detected a stable proportion of 0.005% soy DNA in the sample, while the proportion of spiked-in soy material in the sausage was 0.0316%, suggesting a matrix-dependent underestimation by a factor of 6. We point out, however, that *qualitative* detection may be the prime goal in allergen analysis [46]. The limits of AFS for allergen detection clearly have to be investigated further.

### Technical considerations and further improvements

Next-generation sequencing methods represent the fastest growing technology worldwide, with ever decreasing cost per analysis (http://www.genome.gov/sequencingcosts/). Applying novel 96-well format multiplex methods for Illumina library preparation (NEXTERA®) and a personal sequencer (MiSeq®) we calculate current sequencing cost (chemistry plus personnel, but excluding the bioinformatic analysis) at roughly 150–200 Euro per sample, which may already now be interesting and feasible for routine screening purposes. Although we produced 100 bp paired-end reads for the KalD sample, the initial results on KLyoA suggest that cost-saving 50 bp single-end reads will probably perform equally well in read mapping. However, shorter reads may pose more problems in database searches, unless the BLAST version is optimized for short query sequences.

**Figure 3:**
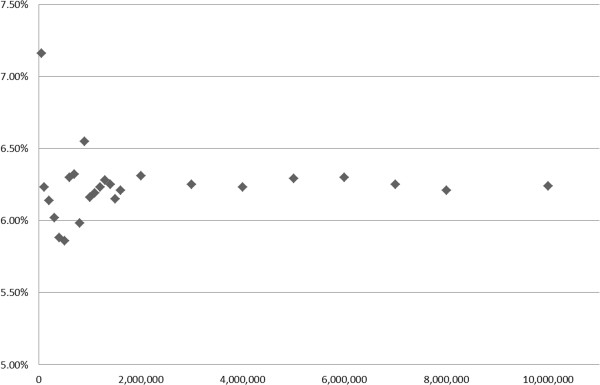
**Determination of the optimal number of sequence reads necessary to obtain accurate quantification results for species components.** The number of sequence reads used in the mapping (x-axis) was plotted against the values of mapping accuracy (y-axis), calculated as the cumulated absolute deviation in% of mapping results versus expected species proportions.

An additional cost saving can possibly be achieved by optimizing the numbers of sequence reads necessary to obtain stable quantification results. To this end, we mapped different numbers of reads, starting with 50 K and multiples thereof up to 10 mio reads, and calculated the sum of deviations (in%) of the observed from the expected species proportions (Figure 3). Deviations decreased with increasing dataset size, but were already close to the optimum at 1 mio reads. Even at 50 or 100 K reads, the sum of deviations was rather moderate, opening the perspective that even very small datasets will still guarantee a reasonable quantification result for the main sample ingredients.

Throughput of samples in time will improve, especially when using the MiSeq® instrument, running only 6 hrs for 50 bp reads. DNA size requirements (>300 bp) and input amounts needed (1 ng) for the NEXTERA XT® protocol [47] are routinely obtained in current PCR-based foodstuff analytics (reviewed in [48]). The slight chance for a wrong allocation of multiplexed samples, which may e.g. be due to erroneous bioinformatic sorting of multiplexing tags, will be substantially reduced by the use of two such tags per sample in the NEXTERA protocol [49, 50]. Another practical problem which has to be adequately addressed is the possible run-to-run carry-over of DNA molecules e.g. due to incomplete removal of residual DNA washing from the sequencing device. Illumina’s technical notes say that this detrimental effect is typically below 0.05% (thus affecting 500 reads in 1 million) and must be controlled by dedicated device maintenance procedures.

On the bioinformatic side, the read-mapping process can already be carried out on standard PCs with 4 GB of RAM using commercial software tools, but is still time-consuming when many reference genomes are inspected. New developments in software programming offer the use of fast and affordable graphics processing units (GPUs) to analyze massive sequence data in reasonable time. To test if such compute unified device architecture (CUDA)-based programs will speed up our pipeline, we compared the novel mapping tool CUSHAW [30] to the standard tool BWA for the time needed for analyze the species proportions of the sausage sample. While the accuracy using CUSHAW appeared somewhat lower than BWA possibly due to algorithmic differences (data not reported), time improvement using CUSHAW was substantial with a 2.0 to 2.6-fold speed-up, depending on the number of threads (one to eight) BWA was allowed to use. CUSHAW thus could cut the time needed for read mapping on a PC roughly by half.

The biggest limitation in our pipeline in terms of time and costs was set by the massive BLAST routines (carried out on our University high-performance cluster) necessary for the metagenomic step. Our *adhoc* calculations suggest that additional costs (ca. 100 EUR) have to be considered, if access to a commercial computing facility is needed. The cautionary tale of the wrongly assembled/annotated *Neisseria* reference genome in our metagenomic step illustrates that the correct interpretation of the BLAST/MEGAN results still requires substantial biological and bioinformatical knowledge. The use of curated sequence database information and/or the application of dedicated repositories containing validated species-specific sequence data (such as bar-coding targets; http://www.barcodeoflife.org/) will greatly simplify this step for non-specialists on the food control side. We wish to point out that a number of highly innovative approaches for the identification (but not necessarily quantification) of species have recently been established in the field of bacterial metagenomics, making use of curated taxon-specific sequence databases (e.g. MetaPhlAn [51]), ultrafast algorithms for sequence pattern recognition (e.g. *k-mer* based methods; [52]) or a probabilistic framework for read assignment to very closely related genomes (e.g. Pathoscope [53]). Integration of these tools is a promising option for further improvement of AFS.

## Conclusion

AFS has the potential to be a valuable method for routine testing of food material and other biosurveillance applications, offering an attractive combination of unbiased screening for all types of ingredients and the possibility of simultaneously obtaining quantifiable results. Since deep DNA sequencing has already revolutionized biological and medical research, it may find its way into routine diagnostics soon. AFS implementation currently requires elaborate knowledge of genomes and bioinformatics, but several strategies are conceivable to further simplify and standardize the approach.

## Electronic supplementary material

Additional file 1: Table S1: Reference genomes used in AFS. (DOC 44 KB)

Additional file 2: Table S2: Mapping results for the reference sausage KLyoA. (DOC 34 KB)

Additional file 3: Table S3: Plant components: spiked in proportions and respective BLAST-hits. (DOC 36 KB)

## Abbreviations

AFS: All food sequencing
KalD: Kalibrator D
KLyoA: Kalibrator Lyoner A
GPUs: Graphics processing units
NGS: Next-generation sequencing.
