# Supplementary material for: All-Food-Seq (AFS): a quantifiable screen for species in biological samples by deep DNA sequencing
Source: BMC Genomics. 2014 Jul 31;15(1):639. doi: 10.1186/1471-2164-15-639 (PMC4131036; doi:10.1186/1471-2164-15-639)
Supplement: Supplementary file 1 — Additional file 1: Table S1: Reference genomes used in AFS. (DOC 44 KB) [file 12864_2013_6336_MOESM1_ESM.doc]

**Table S1 - Reference genomes used in AFS**

| **Species** | **Version** | **NCBI-Accession** |
| --- | --- | --- |
| *Bos taurus* | V 3.1 UMD (Zimin and others 2009) | PRJNA33843 |
|  | V 4.6.1 Baylor College of Medicine (The Bovine Genome Sequencing and Analysis Consortium and others 2009) | PRJNA13366 |
| *Bubalus bubalis* | V 2 BBU (Joshi and others 2009) | PRJNA40113 |
| *Equus caballus* | V 3 (Wade and others 2009) | PRJNA19129 |
| *Escherichia coli* | V 1 (Ferenci and others 2009) | CP001396 |
| *Gallus gallus* | V 2.1 (International Chicken Genome Sequencing Consortium 2004) | PRJNA10808 |
| *Glycine max* | V 1 (Schmutz and others 2010) | PRJNA48389 |
| *Homo sapiens* | V 37 (International Human Genome Sequencing Consortium 2001) | PRJNA168 |
| *Listeria seeligeri* | V 1 (Steinweg and others 2010) | NC_013891.1 |
| *Meleagris galloparvo* | V1 (Dalloul and others 2010) | PRJNA62397 |
| *Mus musculus* | V 9 (Church and others 2009) | PRJNA169 |
| *Neisseria gonorrhoeae* | V 1 (Chen and others 2011) | PRJNA46993 |
| *Oryctolagus cuniculus* | V 2 (Lindblad-Toh and others 2011) | PRJNA42933 |
| *Oryza sativa* | V 4 (Rice Annotation Project 2008) | PRJNA122 |
| *Ovis aries* | V 2 Chromosomes (Archibald and others 2010a) | PRJNA169880 |
|  | V 1 Mitochondrium (Hiendleder and others 1998) | NC_001941.1 |
| *Rattus norvegicus* | V 4 (Rat Genome Sequencing Project Consortium 2004) | PRJNA12455 |
| *Shigella boydii* | V 1 (Yang and others 2005) | PRJNA58215 |
| *Sus scrofa* | V 3.1 (Archibald and others 2010b) | PRJNA13421 |
| *Triticum aestivum* | V 1 Mitochondrium (Ogihara and others 2005) | NC_007579.1 |
|  | V 1 Plastides (Ogihara and others 2002) | NC_002762.1 |
| *Zea mays* | V 1 Chromosomes (Schnable and others 2009) | PRJNA10769 |
|  | V 1 Chloroplasts (Maier and others 1995) | NC_001666.2 |
|  | V 1 Mitochondrium (Clifton and others 2004) | NC_001400.1 |
