# Supplementary material for: All-Food-Seq (AFS): a quantifiable screen for species in biological samples by deep DNA sequencing
Source: BMC Genomics. 2014 Jul 31;15(1):639. doi: 10.1186/1471-2164-15-639 (PMC4131036; doi:10.1186/1471-2164-15-639)
Supplement: Supplementary file 2 — Additional file 2: Table S2: Mapping results for the reference sausage KLyoA. (DOC 34 KB) [file 12864_2013_6336_MOESM2_ESM.doc]

1. **Table S2 - Mapping results for the reference sausage KLyoA**

Quantitative determination of the main species components of the “KLyoA” reference sausage [9]. The AFS-quant procedure was applied with 2 rounds of decreasing mapping stringency (0-1 mismatches). The dataset contains 91,027 Illumina 50 bp single-end reads only. “Difference abs.” shows the difference between the proportion of reads as determined by AFS (“proportion”) relative to the expected amounts existing in the sample (“target value”). The avian ingredients are correctly determined with high quantification accuracy. False positive results are observed for water buffalo, horse and sheep and stay below the 1 % threshold.

| **Species** | **Target value [%]** | **Proportion [%]** | **Difference abs. [%]** | **Difference rel. [%]** |
| --- | --- | --- | --- | --- |
| **Cattle** | 14.00 | 15.28 | 1.28 | 9.14 |
| **Waterbuffalo** | 0.00 | 0.45 | 0.45 | n.a. |
| **Horse** | 0.00 | 0.08 | 0.08 | n.a. |
| **Pig** | 80.00 | 78.20 | 1.80 | 2.25 |
| **Chicken** | 0.50 | 0.72 | 0.22 | 44.00 |
| **Turkey** | 5.50 | 5.19 | 0.31 | 5.64 |
| **Sheep** | 0.00 | 0.08 | 0.08 | n.a. |
| **Total** | 100.00 | 100.00 | 4.14 | 61.03 |
