# Supplementary material for: All-Food-Seq (AFS): a quantifiable screen for species in biological samples by deep DNA sequencing
Source: BMC Genomics. 2014 Jul 31;15(1):639. doi: 10.1186/1471-2164-15-639 (PMC4131036; doi:10.1186/1471-2164-15-639)
Supplement: Supplementary file 3 — Additional file 3: Table S3: Plant components: spiked in proportions and respective BLAST-hits. (DOC 36 KB) [file 12864_2013_6336_MOESM3_ESM.doc]

**Table S3 - Plant components: spiked in proportions and respective BLAST-hits**

List of plant species spiked into the KalD sausage. The relative proportions of the plant ingredients are given, as are the number of BLAST hits, which were recorded by the metagenomic step in AFS.

| **family** | **scientific name** | **trivial name** | **proportion in [%]** | **counted blast-hits** |
| --- | --- | --- | --- | --- |
| Anacardiaceae | *Anacardium occidentale* | cashew nut | 0.003 | 1 |
|  | *Pistacia vera* | pistachio | 0.01 |  |
| Apiaceae | *Apium graveolens* | celery | 0.01 | 17 |
| Betulaceae | *Corylus avellana* | hazelnut | 0.1 | 3 |
| Brassicaceae | *Sinapis alba* | mustard | 0.1 | 449 |
| Fabaceae | *Arachis hypogaea* | peanut | 0.032 | 62 |
|  | *Glycine max* | soybean | 0.032 |  |
|  | *Lupinus spec.* | lupines | 0.316 |  |
| Juglandaceae | *Juglans regia* | walnut | 0.316 | 9 |
| Pedaliaceae | *Sesamum indicum* | sesame | 0.003 | 2 |
| Rosaceae | *Prunus dulcis* | almond | 0.01 | 6 |
